# A long view of social mobility in Scotland and the role of economic changes

**DOI:** 10.1111/1468-4446.13162

**Published:** 2024-11-17

**Authors:** Lindsay Paterson, Fangqi Wen, Richard Breen, Cristina Iannelli, Jung In

**Affiliations:** ^1^ School of Social and Political Science University of Edinburgh Edinburgh UK; ^2^ Department of Sociology Ohio State University Columbus Ohio USA; ^3^ Nuffield College University of Oxford Oxford UK; ^4^ Moray House School of Education and Sport University of Edinburgh Edinburgh UK; ^5^ Department of Sociology University of Copenhagen Copenhagen Denmark

**Keywords:** absolute social mobility, agricultural employment, economic change, education, industrial employment, loglinear modelling, relative social mobility

## Abstract

Changes in the social mobility of men in Scotland between the late‐19th and the late‐twentieth century are examined using new individual‐level data from nineteenth‐century censuses, linking records of men aged 0–19 in 1871 to their records in 1901, and then comparing their patterns with the social mobility of men aged 30–49 in 1974 and in 2001 as recorded in social surveys at these dates. The extent of social mobility in the nineteenth century was large. In particular, the social origins of people in the highest classes—the salariat—were very varied, indicating a society that was more open than is sometimes supposed. There was a slow growth in social mobility between then and 2001. In both periods, class inheritance—sons in the same social class as their father—was strongest in the economically declining sectors, which were agriculture and fisheries in 1901 and industry in 1974 and 2001. In the 1901 data, however, the transition to a non‐agricultural economy induced strong outward mobility from agriculture.

## 1 INTRODUCTION

Over the past 3 decades there has been a growing interest in long‐term changes in intergenerational social mobility, with a particular focus on how much mobility there was in the 19th century and how it has changed since then (e.g., Song et al., 2020). These studies provide a context in which to interpret current levels and trends in mobility. Cross‐national comparative studies (Breen, 2004; Breen & Müller, 2020; Erikson & Goldthorpe, 1992) have found that, although, in many European countries and the United States, mobility increased in the decades following the World War II, this increase has stalled in more recent years and for more recent birth cohorts. That was because the driving force for post‐war mobility was not deliberate policy but rather general economic change. In particular, the growth of the service economy induced upward occupational mobility through growing numbers of relatively high‐status jobs and a shrinking need for low‐status manual workers. When that expansion slowed, the upward mobility which it had brought about correspondingly declined. This is true of both absolute mobility and, in many European countries, relative mobility, sometimes called social fluidity: more room at the top meant that people who were born outside the more advantaged classes had greater opportunities of entering them while not threatening the ability of those who were born into those classes to remain in them.

This leads us to wonder whether the current stagnation of mobility rates is simply returning us to a situation that held before the mid‐20th century upsurge or whether there is, notwithstanding this, greater mobility nowadays than in the past. In this paper we address that question using data from Scotland which cover the whole of the 20th century. In particular, we analyse how social mobility patterns differed across cohorts of men which experienced major economic changes—namely industrialisation and the growth of the service economy—and how, in 1901, mobility patterns differed across geographical areas with different economic characteristics. A number of studies have documented patterns of social mobility in Scotland in the second half of the twentieth century, and the Scottish experience of social mobility has often been analysed as an informative case study in wider debates about social mobility internationally (e.g., Erikson & Goldthorpe, 1992; Iannelli & Paterson, 2006; Paterson and Iannelli, 2007; Payne, 1987; Hope, 1984). The general conclusion of these comparisons is that Scotland has been quite typical in its rates of both absolute and relative mobility, and in the trends in these since 1945. But, as yet, there is no comprehensive national study of social mobility in Scotland from the late 19th century. Here we present the first such study, using survey data from the late 20th century and linked census data from the late 19th century. Scotland is an interesting case because of its distinctive features compared to England which may have shaped social mobility patterns between the late 19th and early 20th centuries: among them, its somewhat later industrialisation, the different employment structure of its agriculture sector, the specificities of its regional variation, and the strong sense of distinctiveness, manifested in the belief that Scottish society was more open and inclusive than English society.

## 2 SOCIAL MOBILITY IN THE 19TH CENTURY

The consensus in the last 2 decades of research in several countries on social mobility in the nineteenth century is that it was more extensive than had been supposed by social scientists, that it increased during that century, and that it increased further during the twentieth century, but at a slower rate (Heath & Li, 2024, pp. 139–147). van Leeuwen and Maas (2010, p. 435) note that the previous consensus had been that ‘the degree of intergenerational mobility is basically stable over time’, as concluded by Lipset and Zetterberg (1959), studying rates of absolute mobility, and by Featherman et al. (1975) on relative mobility. Although some research continues to find basic stability in some regions (e.g., Zijdeman (2008) for Zeeland), it seems now that mobility was not negligible in most of the industrialised parts of nineteenth‐century Europe. For example, Miles (1993) analysed marriage registers in England and Wales for the period 1839–1914, and found increasing rates of mobility. Grusky (1986) found growing relative mobility in the United States as the country industrialised. Ganzeboom et al. (1989) found increasing relative mobility in several countries. Lambert et al. (2007) drew together a wide range of contemporary sample surveys of Britain (as distinct from studying the past through birth cohorts constructed from later surveys) and found a slowly weakening correlation over time between the social status of fathers and sons (and also daughters). Fitting a regression line to this trend, they estimated that the rate of decline of the father—son correlation from 1900 to 2000 was about 0.01 per decade.

Until recently, the main source of evidence about nineteenth century social mobility in European societies has been marriage registers but these have some obvious shortcomings, not least that they exclude people who never married or married outside the established churches and that parental occupation is measured when a person marries rather than when they were growing up. An alternative source of population data on mobility in some countries is the census. Long (2005, 2013) was a pioneer in such work on England and Wales. He studied mobility both between fathers and sons, and within the sons' careers, using linked census data on fathers and sons for the periods 1851‐81 and 1881–1901. He concluded that ‘intergenerational social mobility was markedly greater than previous estimates have indicated’ (Long, 2013, p. 2). These rates were also lower than had been found in research on the late‐twentieth century (ibid.: 12–14): the increase in the total proportion who had been mobile (up or down) in the century to 1972 was equivalent to about 0.01 per decade. The findings of high and rising rates of mobility in the nineteenth century confirms contemporary observations, as Miles (1993) notes.

While industrialisation and urbanisation are often pointed to as possible explanations for increasing mobility rates, the other explanation that has been offered—and that was cited contemporaneously—is education. However, although noting some association of the increase in mobility in England and Wales with the advent of compulsory primary schooling in 1870, Long (2013: 29) comments that the association is weaker than might be expected. The policy change did not produce a step‐change in rates, and so may have been no more than a new means of facilitating what was under way for other reasons relating to change in the occupational structure.

## 3 THE SCOTTISH CASE

Scotland was thoroughly typical of all these processes of occupational change, especially by comparison with England and Wales. However, Scotland's industrial revolution started several decades later than that in England (abruptly in the first 2 decades of the nineteenth century rather than more slowly in the second half of the eighteenth century (Devine, 2000, pp. 105–123)). Scotland rapidly caught up. As explained below, we use data on men aged 30–49 in 1901, 1974 and 2001. They would have entered employment approximately in the 1870s and 1880s, the 1940s and 1950s, and the 1970s and 1980s. Our results can therefore be interpreted in relation to the structure of employment in these three periods.

By the second half of the 19^th^ century, Scottish employment was coming to be dominated by manufacturing and associated services. The share of male employment in agriculture in Scotland declined: from 30% in 1851 to 19% in 1881 and 13% in 1911 (Treble, 1990, pp. 195–196). Manufacturing employment was stable at around 40% over that half century, and the share in services (including transport) rose from 18% through 23% to 28%. The decline in agricultural employment mainly affected the predominantly rural areas in the south, the north‐east and the north.

These changes would themselves be expected to stimulate mobility, just as the transition away from manufacturing did a century later. There was also mobility within agriculture—routes from ‘farm labour’ to the class of self‐employed farmers. This was because the employment structure of Scottish agriculture at this time was not as firmly divided into large capitalist farmers and land‐less labourers as farming in the south of England had been since earlier in the nineteenth century (Hobsbawm & Rudé, 1969). Carter (1979), noting that the southern English experience did not accurately describe Scottish agricultural employment outside the Lothians, traced the continuing strength in the north‐east of the class of substantial peasants (ibid.: 95). There were routes into this self‐employed farming class from occupations that would be classified as agricultural labour. In the south west, there was a category of skilled farm labour which essentially acted as contractor to large farmers in the management of livestock, and which provided routes into the ownership or tenancy of farms (Campbell, 1984). Thus, as Carter (1979: 109) points out ‘for most men, farm service was not a career in itself but a stage in a career that started outside farm service and would finish outside’.

There were three important ways in which the changes to the structure of employment might have created scope for social mobility from the late ninenteenth century. The most obvious—and the one that was most shared with the rest of Britain and with other countries that were experiencing similar economic changes—was the growth of the service economy. The second was the last stages of the transition from a rural economy to an industrial one, attracting labour from the periphery into the growing urban centres. Again, this was familiar from elsewhere. The third, however, was a consequence of the continuing vitality of agriculture as the agricultural recession of the 1870s and 1880s was left behind. Treble (ibid.: 167‐8) also notes that the decline of agricultural labour in Scotland was due more to the pull of the cities than to any decline of the labour requirements of farms. So unemployment did not rise in agricultural areas, and wages rose.

In our paper, we link social mobility to the economic characteristics of broad areas by relating the employment structure of Scottish counties in 1901 to the social mobility patterns of those living in the counties. Montt and Maas (2015) found that, in Britain in the nineteenth century, living in an urban area was generally associated with men's reaching a higher social status (controlling for their father's status and their own education) than for men living elsewhere. Schulz et al. (2015) showed similarly, for the Netherlands between 1812 and 1922, that municipalities which had high levels of modernisation were associated with a higher status of men's employment at the beginning of their careers. Lippényi et al. (2015) found, for Hungary between 1870 and 1950, that, among men whose father was in a non‐manual class, areas with higher levels of industralisation had greater social mobility than elsewhere. Thus our paper treats Scotland as a case study which can provide further insights on the role of structural changes in the economy for social mobility.

The other reason to be interested in the long‐term changes in intergenerational social mobility in Scotland is somewhat more specialised, though also with wider resonance. An ideology of social mobility has been described as the dominant Scottish myth (in an anthropological sense) from the 18th to at least the middle of the twentieth century. According to this, the Scottish social structure was more open than elsewhere, although in practice that comparison was usually with England (McPherson, 1984). The claim was that Scotland was closer to Turner's model of contest mobility, in contrast to England's sponsored mobility (Turner, 1960). In Turner's analysis, this would then place Scotland close to the United States, a hypothesis that was investigated most thoroughly by Hope (1984). The iconic social figure in that story was the ‘lad o pairts’, a Scots‐language term for a boy with a broad range of abilities and interests who was picked out by the parish school teacher for special tutoring in the advanced subjects that would enable him to compete for entry to university and for a bursary to support his studies there (Anderson, 1983, 1995). The scope for this was in the national system of parish schools that had been established, in principle, after the Reformation of 1560, and in practice had achieved universal coverage outside the Highlands by the mid‐eighteenth century. When this system was taken over by the state after 1872, there was unprecedented scope for the fullest flourishing of the myth (as also for its gradual extension to girls). Anderson (1983) and McPherson (1984) note that there were enough prominent Scots who had indeed come through this route to sustain belief in the myth until well into the twentieth century.

Anderson (1983, p. 158) notes that the myth had two versions, both dependent on education. One was rural, epitomised by the north east, which led through academic courses to a professional career. The other was urban, giving access to commercial opportunities. But restricting the scope of the myth were the economic realities facing most households. Despite the prospect of bursaries, few boys could afford to remain in education beyond age 14. That was the official minimum leaving age from 1883, but exemption for reasons of employment were quite easy to obtain until 1901. So education might in principle be a route to opportunity, but in practice might be unaffordable simply because families could not forgo the earnings that adolescents might bring.

Scotland as a case study, and Scotland as a land of opportunity, thus give research questions that guide our analysis here. In each of these, the meaning of social mobility might be both absolute and relative

1. Did Scottish social mobility slowly increase in the twentieth century?
2. Did Scotland have the same pattern of quite extensive social mobility in the late‐nineteenth century as has been observed in recent research on other societies?
3. Were the rates of social mobility in the late‐nineteenth century related to the structures of employment where men were living in 1901?
  - Specifically, did the more industrial areas have the greatest mobility?
  - Or was mobility greater in the residual agricultural areas (as might be suggested either by the persisting Scottish myth, or by the very fact that such areas were losing labour to the non‐agricultural economy)?
4. Was there any evidence that education played any part in shaping the patterns of social mobility in 1901?

## 4 DATA AND METHODS

We use data from three sources. The first of these is the digitised individual household returns from the 1871 and 1901 censuses of population.^1^ We focus on men aged 0–19 in the 1871 returns (when we can find the occupation of their father or head of the household) and link them to their own occupational record in the 1901 census when they were aged 30–49. The linkage is described in detail by Wen et al. (2022). It is based on exact matching: that is, records in the two censuses are inferred to be of the same man if they agree on ‘first name, surname, age, and county and parish of birth’ (ibid.: 6). Only about 20% of potential matches were made (i.e., as a proportion of men aged 0–19 in 1871 who had not emigrated or died). This resulted in 96,046 cases. Notice that we cannot use this method to link women across censuses because they usually changed their surname at marriage to that of their husband. Weights were calculated to adjust the data to the distribution of this age group of men in the 1901 census. The weights were nearly symmetrical around a mean of 1 (the median being 0.93), and did not vary much: the lower and upper quartiles were 0.84 and 1.05, and the standard deviation was 0.29. The symmetry and quite tight clustering of weights suggest that the achieved sample was quite representative of the population of 0‐19‐year‐old men who were resident in Scotland in 1901 and who had been born there. After omitting cases that had missing data on the weights, on the respondent's occupation in 1901, or on the father's occupation in 1871, the sample size was 84,057.

Linking across censuses in this way potentially leads to two kinds of error: type I, or false positives, when we incorrectly link the records of different persons believing them to be of the same person, and type II, or false negatives, when we fail to find a link for a person who was actually present in both censuses. It is widely recognised that the former are the more problematic for the study of mobility because they tend to inflate the amount of mobility that we observe. Exact matching, because it is quite a stringent criterion, minimises the number of type I errors but, compared to other methods, it probably leads to more type II errors. This tends to make the resulting dataset less representative of the population, but we seek to mitigate this problem by the reweighting described above.

The second source of data is the 1974 Scottish Mobility Survey (UK Data Service study number 981) which covered men resident in Scotland in that year (Payne, 1987). We use respondents who were aged 30–49. The sample size was 2,116, which fell to 1669 when missing data were omitted. No weights are available for this data set, but the response rate of the survey was 82% (Payne, 1987, p. 197).

The third source is the 2001 Scottish Household Survey (UK Data Service study number 4642). That survey has been carried out annually since 1999 on behalf of the Scottish Government. In the 2001 survey a module of questions on parental occupation was included (Iannelli & Paterson, 2006). Again, the analysis is restricted to men aged 30–49. Weights are used to match the distribution to census data in 2001. The sample size was 1,693, which fell to 1625 when cases with missing data were excluded. Repeating the analysis using the unweighted 2001 data gave very similar results to those reported below.

The social class of father and son is measured by the seven‐class version of the Goldthorpe scheme (Goldthorpe, 1980), as shown in Table 2. For the 1901 data, the occupation data for fathers, taken from the 1871 census, and for sons, taken from the 1901 census, were recoded into the Goldthorpe scheme by Wen et al. (2022) using ISCO68 provided in the IPUMS‐International database which in turn was derived from the HISCO codings generated by the Integrated Census Microdata project, I‐CeM (Wen et al., 2022).^2^ HISCO is the Historical International Standard Classification of Occupations which allows historical descriptions of occupations to be translated into common sociological systems of classification (van Leeuwen et al., 2004). In the 1974 and 2001 surveys, information about father's occupation was recalled by respondents and recorded in the categories that form the basis of the Goldthorpe scheme. The reason to retain the full seven class categories was to allow attention in 1901 to classes IVc and VIIb, recording occupations in agriculture, forestry and fishing (which we refer to for brevity as simply agriculture), in which 17% of sons and 28% of fathers were found. These categories had become negligible by 1974, and so for comparison over time they are combined with other categories into a five‐class schema, putting self‐employed in agriculture, IVc, together with the other self‐employed class, IVa,b, and combining agricultural workers, VIIb, with semi‐skilled and unskilled manual workers, VIIa.

Part of the 1901 analysis uses data also from two other sources, each relating to the county where the sample member was living in that year. One source describes the structure of male employment in the county, obtained from the published results of the 1901 census. The other source is an index of participation in primary schooling in the county drawn from the annual report of the Scotch Education Department (1896). In the absence from the census data of any information on the sample member's own education, this is the only way to test whether education might have been relevant to the patterns of social mobility.

We have no information about men who emigrated from Scotland because they were not included in the 1901 census nor in the 1974 and 2001 populations from which our samples were drawn. Emigration out of the UK fluctuated throughout the twentieth century, affected by economic conditions in the UK and in the main receiving countries of North America and Australasia, but, as a proportion of the Scottish population, it probably was similar in the late‐nineteenth century and a century later. At the time of the 1901 data, the rate of annual outward passenger movements was about four per 1000 population (Anderson, 2018, p. 142). That was similar to the rate of movement out of the UK in the 1990s (ibid.: 147). Emigration from Scotland has tended to be meritocratic throughout the past two centuries, and it has also often been chain migration, which is the tendency of people to follow successful migrants from their own extended families (Paterson and Iannelli, 2007, p. 355). Migrants are likely to have been upwardly mobile (presumably they emigrated for that reason) and their exclusion from our analysis may lead to an under‐estimation of the overall rates of upward mobility. Having said that, the lack of data on those who emigrate makes it difficult to know how this affected the social mobility of those who did not migrate, or how, if the migrants had not migrated, social mobility in Scotland might have been different.

In the analyses that follow we consider absolute and relative mobility separately, in each case focusing first on changes over time and then on 1901.^3^ Absolute mobility is simply the description of, for example, the percentage of men of a particular class of origin who have been upwardly mobile. This is sociologically interesting in itself, but it fails to distinguish between mobility induced by the changing class structure—for example, by the growth of the professional class and the shrinking of the manual classes—and changes in the relative chances that people from different class origins will enter, for example, the professional class. Relative mobility may be thought of loosely as the comparison of rates of mobility of men of different classes of origin. We focus in particular on a commonly used measure called ‘unidiff’, which allows us to compare relative mobility across a set of mobility tables by providing, for each table, a single number that summarises the strength of the relationship between class origins and destinations (see Erikson & Goldthorpe, 1992, pp. 91–92).

We look in more detail at 1901 using comparisons across the 33 Scottish counties to address questions 3 and 4, above. The models were estimated by the packages ‘glm’ and ‘gnm’ in R. A fuller summary of the modelling of mobility tables in R is provided by Friendly (2023).

## 5 RESULTS

### 5.1 Comparison over time

#### 5.1.1 Absolute mobility

Rates of absolute mobility increased between 1901 and 2001; as Table 1 shows, just over half of men in 1901 occupied a destination class different to their origin, while, in 2001, two‐thirds did. Table 1 also shows that this growth in mobility was entirely due to greater upward mobility, with rates of downward mobility remaining constant.

**TABLE 1:** Absolute social mobility, by date.

Column percentages	Date		
	1901	1974	2001
Mobile	52	64	67
Upwardly mobile	30	42	45
Downwardly mobile	22	22	22

*Note*: Percentages are weighted in 1901 and 2001.

The increase in upward mobility was driven by structural change, which was particularly rapid in the final decades of the 20th century. Table 2 shows the distribution of origins and destinations at the three dates. There was a substantial rise in the proportion of destinations in the category high‐ and low‐grade professional and managerial occupations (I,II), evident already in 1974 and doubling in the next 3 decades. There was also a large fall in the share of manual supervisors and skilled workers (V,VI), and semi‐skilled and unskilled manual workers (VIIa,b), although that includes a rise in the semi‐ and unskilled proportion in 1974 before a large fall. The two agricultural categories dwindled over the century. The impact of this structural change can be summed by the index of dissimilarity, capturing the difference between the origin and destination distributions in each year: it grew from 0.12 in 1901, to 0.16 in 1974 to 0.23 in 2001, indicating an increase in social mobility.^4^ In 1901 the index was mostly driven by the decline, between origins and destinations, in the share of self‐employed in agriculture (IVc) and agricultural workers (VIIb). In 1974 the index was driven largely by the increase in the high and low‐grade professionals and managers (class I,II) and the decline in skilled manual workers (class V) and semi‐skilled and unskilled manual workers (class VI,VIIa). In 2001 the index was wholly driven by the growth of the class of high and low‐grade professionals and managers and compensating declines spread across all the other classes (except for skilled manual workers).

**TABLE 2:** Origin and destination, 1901, 1974, and 2001.

Column percentages	1901		1974		2001	
	Origin	Destination	Origin	Destination	Origin	Destination
High and low‐grade professional and manager (I, II)	4.9	7.3	9.5	23.9	27.4	46.9
Routine non‐manual (III)	1.7	5.2	6.4	7.8	17.1	8.9
Self‐employed (not agriculture) (IVa,b)	6.6	8.7	7.7	8.0	7.1	4.8
Self‐employed (agriculture) (IVc)	11.4	5.4	2.6	1.0	1.5	<0.1
Manual supervisor and skilled manual (V,VI)	38.4	41.1	37.6	31.4	22.5	26.3
Semi‐skilled and unskilled manual (VIIa)	20.3	21.2	30.9	25.6	22.9	13.1
Agricultural worker (VIIb)	16.9	11.1	5.3	2.2	1.5	0.1
Dissimilarly index	0.12		0.16		0.23	
Sample size	84,057		1669		1625	

*Note*: Percentages are weighted in 1901 and 2001; sample sizes are unweighted. 1974 and 2001 are male respondents aged 30–49 in these survey years. ‘Agriculture’ includes fishing and forestry. Omits cases for which occupation could not be classified (11% in 1871–1901, 3.1% in 1974 and 0.5% in 2001).

Although changes in the share of agricultural work account for most of the dissimilarity index in 1901, agriculture was not what most men, or their fathers, did. Self‐employed in agriculture and agricultural workers made up 28% of origins but only 17% of destinations, compared with 38% and 41% for skilled manual workers. A striking contrast is between class I&II, which accounted for very few men in 1901 but dominated in 2001, and self‐employed in agriculture and agricultural workers, who were common in 1901 but had virtually disappeared 100 years later. Skilled, semi‐skilled and unskilled manual workers accounted for more than half the male workforce in 1901 and 1974 but less than 40% by 2001.

#### 5.1.2 Relative mobility

In the following analyses we are concerned with the competition among people from different origins to acquire a position in one destination class rather than another, also called social fluidity. A log‐linear model of constant social fluidity, which posits change over years in the origin and destination marginal distributions but an unchanging association between origins and destinations, fails to fit the three tables by some considerable distance, returning a deviance of 241 with 32 degrees of freedom (Table S1 in the Supporting Information). We conclude, therefore, that social fluidity, or relative mobility, was different in 1901, 1974 and 2001.

The unidiff model, which allows for differences in social fluidity, returns a residual deviance of 183 with 30 degrees of freedom. It is thus a statistically significant improvement on the model of constant social fluidity, reducing the residual deviance by 58 for the use of two degrees of freedom, even though it does not fit the data according to the usual chi‐squared criterion. The unidiff model hypothesises a common pattern of log odds ratios in the three tables and captures differences by allowing these log odds ratios to be uniformly larger or smaller in each table. In our case, setting the unidiff parameter for 1901 on the log‐odds scale to a fixed value of zero, the estimates for 1974 and 2001 are −0.24 (standard error = 0.073) and −0.48 (s.e. = 0.086), showing a weakening association between origins and destinations and therefore increasing social fluidity over the 20th century.^5^

Using the five‐class scheme, the deviations from the constant association model illustrate where the greater fluidity in the later data, especially 2001, arose (Table S2 in the Supporting Information). In 2001, all but one of the five residuals on the diagonal are less than −1.96, showing that class stability is less than would be predicted from earlier years. The exception is in the highest class, the extent of self‐recruitment to which is similar in all 3 years. Moreover, in 2001 all but one of the off‐diagonal residuals from that model which are greater than 1.96 in absolute magnitude involve the two classes that were contracting in size, those that involve skilled, semi‐skilled and unskilled manual workers. There is more movement out of these classes than would be predicted from earlier years, but also more movement into them. With the benefit of the hindsight provided by the 2001 data, the 1974 residuals may be seen as transitional towards these patterns in the sense that there was lower‐than predicted stability in all but the highest and the self‐employed classes.

In the light of these patterns of the residuals, it is informative to fit a topological model that posits constant social fluidity except for change in cells on the main diagonal, in other words modelling only inheritance of class position (see Bukodi et al. (2016) for an explanation of this type of models). This gave a residual deviance of 54.7 on 22 degrees of freedom, an approximately three‐quarters reduction from the constant social fluidity in Table S1. The resulting parameter estimates are shown in Table S3. These estimates measure the change in class inheritance compared to 1901. All but three of the estimates are negative, meaning a reduction of inheritance, consistent with the residuals shown in Table S2. The exceptions are the professional and managerial class in both 1974 and 2001, showing an increasing tendency for the sons in that class to remain in their father's class. There is also a positive estimate for the self‐employed class in 1974, but the corresponding parameter in 2001 is negative.

Therefore the increase in mobility over the century is mostly driven by declining class inheritance which has been strong enough to counteract the increasing inheritance in the professional and managerial class.

### 5.2 Absolute and relative mobility in 1901

A remarkable feature of the outflow matrix for 1901 shown in Table 3 is its dominance by flows into skilled, semi‐skilled and unskilled manual work. The class of supervisors and skilled manual workers was the most likely destination for men of all origins except for self‐employed in agriculture and agricultural workers. The outflows into agriculture from non‐agricultural origins were negligible, as were the outflows into the class of agricultural workers, except among those of agricultural origins. These trends all reflect what we already saw—the decline, between origins and destinations, in agriculture, and the dominance of manual work, especially skilled manual work, in origins and destinations. As a result, when we examine inflows (Table 4), we see that the most likely origin of men in every class (again, with the exception of the agricultural classes) was the skilled manual class, followed by the semi‐ and unskilled class. Despite the strong tendency for sons to follow their fathers into the same class (as we show below), the small size of the professional and managerial class in origins meant that this destination was very heterogeneous in where its occupants came from. Only 17% came from the same class, and almost half (46%) came from the non‐agricultural working class.

**TABLE 3:** Outflow percentages (1901).

	Destination							Sample size
Row percentages Origin	High and low controllers	Routine non‐manual	Self‐employed (not agriculture)	Self‐employed (agriculture)	Supervisor and skilled manual	Semi‐skilled and unskilled manual	Agricultural worker	
High and low‐grade professional and manager	25	12	10	2	30	18	3	4245
Routine non‐manual	16	17	12	2	30	19	3	1452
Self‐employed (not agriculture)	11	10	26	2	31	18	3	5616
Self‐employed (agriculture)	7	4	6	31	16	13	22	10,036
Supervisor and skilled manual	6	4	7	1	60	18	3	31,859
Semi‐skilled and unskilled manual	6	5	8	1	42	33	5	16,579
Agricultural worker	5	3	6	6	23	22	35	14,270

*Note*: ‘Agriculture’ includes fishing and forestry.

**TABLE 4:** Inflow percentages (1901).

	Destination						
Column percentages Origin	High and low controllers	Routine non‐manual	Self‐employed (not agriculture)	Self‐employed (agriculture)	Supervisor and skilled manual	Semi‐skilled and unskilled manual	Agricultural worker
High and low‐grade professional and manager	17	11	6	2	4	4	1
Routine non‐manual	4	5	2	1	1	2	<0.1
Self‐employed (not agriculture)	10	12	20	2	5	6	2
Self‐employed (agriculture)	11	10	8	66	4	7	23
Supervisor and skilled manual	30	33	32	8	56	33	11
Semi‐skilled and unskilled manual	16	19	19	5	21	32	9
Agricultural worker	11	10	12	17	9	17	54
*Sample size*	7344	5169	8128	5560	33,261	15,446	9149

*Note*: ‘Agriculture’ includes fishing and forestry.

Table 5 reports the goodness‐of‐fit of several models fitted to the 7‐by‐7 mobility table from the 1901 data. Because of the very large sample size we are unlikely to find a model (other than the saturated model) that fits the data according to the conventional chi‐squared test criterion. We therefore focus on how much of the residual deviance from the model of independence we can account for using different models of the association of origin by destination. The model of independence of origins and destinations returns a very large deviance. Quasi‐perfect mobility is defined as fitting the cells on the main diagonal of the mobility table exactly but retaining the independence assumption for the off‐diagonal cells. The deviance of this model is large in absolute terms but much smaller than that of the independence model: when we add the quasi‐perfect‐mobility terms to the independence model, the residual deviance is reduced by 79%. This strongly suggests that the tendency for sons to follow their fathers into the same class explains a good deal of the overall association between origins and destinations. Table 6 shows the quasi‐perfect‐mobility parameters, also called ‘class inheritance’. All of them are statistically significantly different from zero, showing that class inheritance operated in all classes, but they are particularly large for self‐employed in agriculture and agricultural workers. The only class for which they are notably smaller is semi‐skilled and unskilled manual workers.

**TABLE 5:** Goodness of fit of models applied to table of origins and destinations in 1901.

Model	Degrees of freedom	Residual deviance	% Reduction from independence model
Independence	36	30,992	–
Quasi‐perfect mobility	29	6865	78.8
Quasi‐perfect mobility plus terms for residuals^a^	25	1302	95.8

^a^ Exchange between professional and managerial classes and routine non‐manual, and exchange between the two agricultural classes.

**TABLE 6:** Diagonal (class inheritance) parameter estimates from quasi‐perfect‐mobility model.

Class	Class inheritance	Standard error
High and low‐grade professional and manager	1.44	0.03
Routine non‐manual	0.84	0.07
Self‐employed (not agriculture)	1.13	0.03
Self‐employed (agriculture)	2.79	0.03
Supervisor and skilled manual	0.91	0.02
Semi‐skilled and unskilled manual	0.32	0.02
Agricultural worker	1.84	0.02

Examining the residuals from the quasi‐perfect‐mobility model we find that the four largest, and the only ones with an absolute value greater than one, are for movement between origins in the professional and managerial class and destinations in routine non‐manual, and vice‐versa, and movement between origins in self‐employed agriculture and destinations as an agricultural worker, and vice‐versa. Thus there was more exchange between the two white‐collar classes (professional and manager and routine non‐manual) and the two agricultural classes than would be expected under quasi‐perfect mobility. The third model reported in Table 5 adds parameters to the quasi‐perfect‐mobility model in order to fit these four residuals exactly (as what is often referred to as a topological model). The deviance is substantially reduced, with this model accounting for 96% of the residual deviance of the independence model.

### 5.3 Variation by county in 1901

A recurring theme of the above analysis of social mobility in 1901 is the distinctive position of the two agricultural classes. Therefore we next take this a step further to consider social mobility in more local contexts, relating it to the overall share of local agricultural employment. The data underpinning this analysis, showing information about the distribution of employment within each of the 33 Scottish counties, can be found in the Supporting Information, Table S4. Our main interest is in the percentages of male employment in the categories ‘agriculture and fishing’ (covering farms, woods, gardens and fishing) (Parliamentary Papers, 1903: xiv). There is a general tendency for the agricultural and industrial percentages of employment to be complementary: there is a correlation −0.95 between these two columns in Table S4.

There is strong evidence that mobility varies across counties: the residual deviance from a model that fits a common pattern of social fluidity (CSF) to all 33 counties is large (2335 on 1152 degrees of freedom) (Table 7). We investigate the remaining variation in two ways. One is by explicitly modelling the cells that have large residuals from the model of constant social fluidity, and the other is by calculating unidiff estimates as a single‐valued summary capturing the relative strength of the origin‐destination in each county.

**TABLE 7:** Models for county and movement between and within and out of the agricultural classes (1901).

	Goodness of fit		
	Df	Deviance	p
Common social fluidity	1152	2335	<0.001
Terms added to Common Social Fluidity model			
(a) mobility between and within agricultural classes	1024	1568	<0.001
(b) mobility out of agricultural classes	1088	1814	<0.001
Unidiff	1120	1885	<0.001

*Note*: Agricultural’ includes fishing and forestry.

In the mobility tables for each of the 33 counties (each table being 7‐by‐7, and so with 1617 cells), there were 73 cells where the positive residual from the CSF model is greater than 1.96—in other words, where there was more mobility, or greater stability, than the average across the counties. Of these, 58 involved moving into or out of one of the two agricultural categories: 20 are cells corresponding to moving out of agriculture, 23 into these, and 15 between them. The details are shown in Figure S1 and Table S4 in the Supporting Information.

**FIGURE 1:**
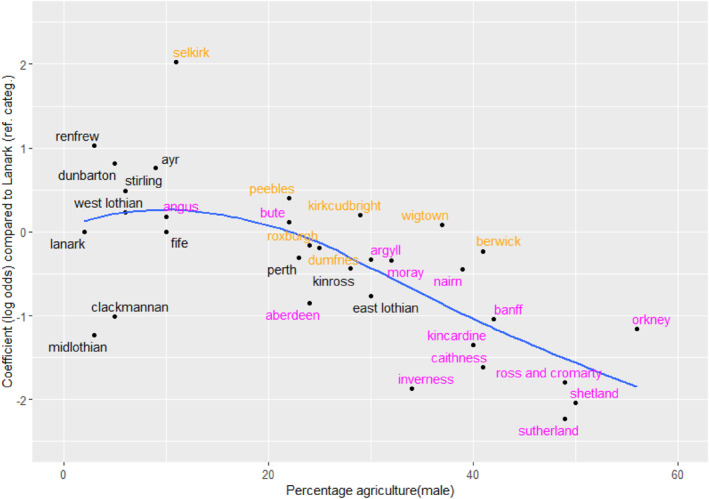
Relationship between stability in self‐employed agriculture and male employment in agriculture (1901). ‘Agriculture’ includes fishing and forestry. From the model in Table 7. The reference category is Lanarkshire (with value 0 on the vertical axis). The line is fitted by locally estimated scatterplot smoothing. The labels in magenta are in the Highlands, Islands and North East; labels in orange are in the south.

We sought to capture those residuals which involve movement within and between the two agricultural categories by adding parameters to the CSF model corresponding to these four cells of the table—one each for stability in the agricultural classes, and one each for movement between these. The goodness‐of‐fit of the resulting model is reported in Table 7, models a and b. The parameters associated with this matrix are allowed to vary across the counties. There are thus in total 128 parameters, because one of the 33 counties has to be a reference category; we set that to be Lanark, as being both the largest county and the county with the lowest share of agricultural employment. The results can be presented by graphing the corresponding parameter estimates against the share of agricultural employment in each county. There was little variation across counties in the movement between the two agricultural classes, but there were clear patterns for stability. Figure 1 shows the pattern of variation for the parameter recording stability in the category of self‐employed in agriculture. High values of the parameter correspond to high stability, and so the graph shows that stability tends to be lower in counties with higher rates of agricultural employment. A similar, but weaker, tendency was found in the stability in the agricultural‐worker class. In other words, where agriculture had shrunk to what might be called a residual sector in a strongly non‐agricultural context (the left‐hand end of this graph), agricultural employment of sons was more strongly tied to agricultural employment of their fathers than in counties where agriculture was still relatively large.

**FIGURE 2:**
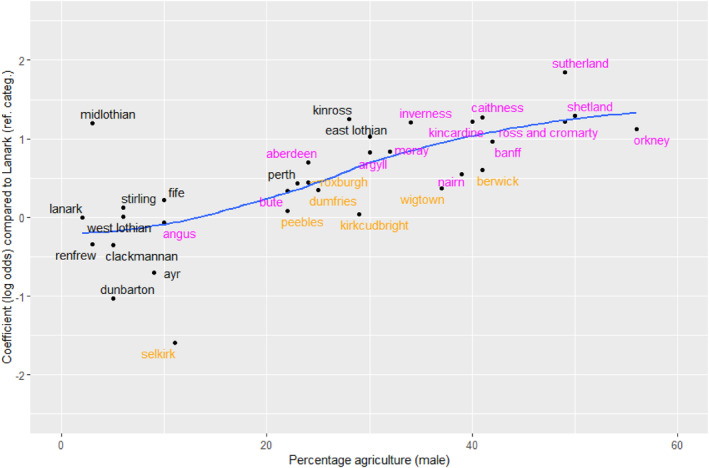
Relationship between movement out of self‐employed agriculture and male employment in agriculture (1901). ‘Agriculture’ includes fishing and forestry. From the model in Table 7. The reference category is Lanarkshire (with value 0 on the vertical axis). The line is fitted by locally estimated scatterplot smoothing. The labels in magenta are in the Highlands, Islands and North East; labels in orange are in the south.

We can investigate what was happening in these more strongly agricultural counties by adding other terms to the CSF model. Here, there are two extra parameters, corresponding to movement out of the two agricultural classes. The goodness‐of‐fit of this model is also reported in Table 7 (model b). The rate of movement out of self‐employed agriculture was greater in the more agricultural counties than in those with minimal agricultural employment (Figure 2). Again, a similar but weaker pattern is seen for movement out of agricultural work. Therefore, although a generally declining sector will eventually be self‐recruiting (as in the least agricultural counties in Figure 1), during the transition that sector will show a large amount of movement outwards.

**FIGURE 3:**
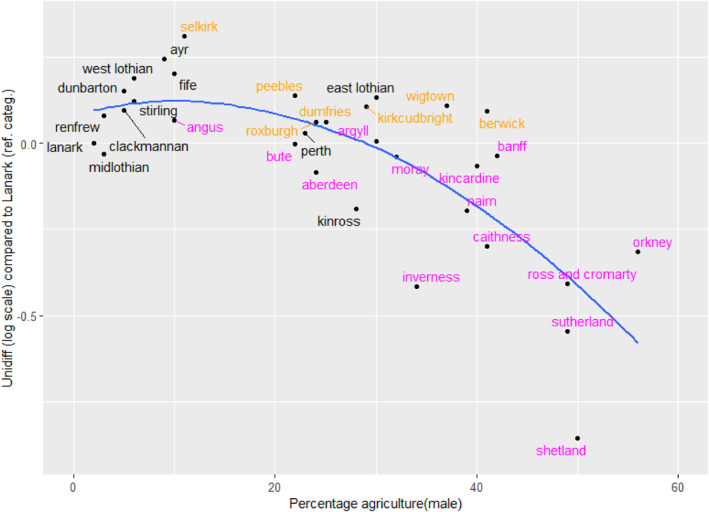
Relationship between unidiff and male employment in agriculture (1901). ‘Agriculture’ includes fishing and forestry. From the model in Table 7. The line is fitted by locally estimated scatterplot smoothing. The labels in magenta are in the Highlands, Islands and North East; labels in orange are in the south.

The unidiff parameters for the counties confirm this. Compared with CSF, it reduces the residual deviance from 2335 to 1885 for the use of 32 degrees of freedom and in Figure 3, the unidiff estimates are graphed against the share of agricultural employment. A low value of unidiff corresponds to greater fluidity. Thus the graph shows that fluidity was largest in counties with the highest levels of agricultural employment, just as the models shown in Figures 1 and 2 would lead us to expect.

The greater fluidity in the agricultural counties of the Highlands and the North East suggests a potential link to educational provision, because by the late‐nineteenth century these counties had relatively high rates of provision, much of it provided by religiously inspired philanthropic endowments (Anderson, 1983, 1995). This further analysis uses, as a broad indicator of the extent of participation in each county, the ratio of the number of children of primary‐school age who attended school in 1895 to an estimate of the size of that age group in 1891 (Parliamentary Papers, 1896, p. 32). This is not strictly a percentage, because the relevant population data are crude estimates, and in any case are not from the same year as the attendance figures. So we refer to this as an index of attendance, shown in the final column of Table S4 in the Supporting Information.

**FIGURE 4:**
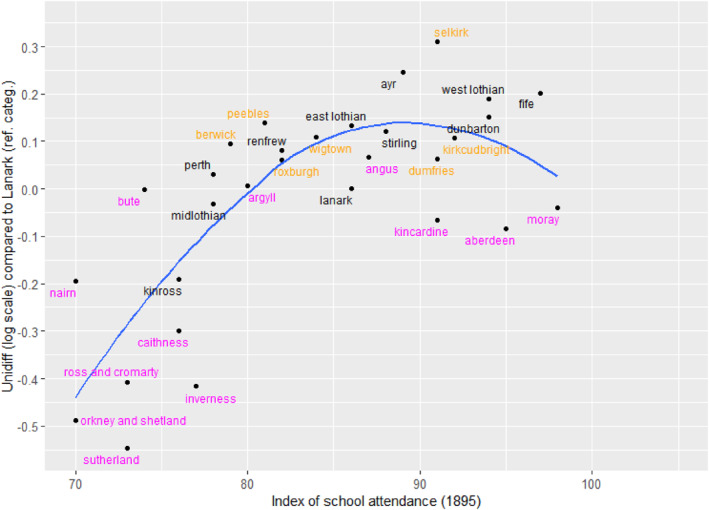
Relationship between unidiff and index of school attendance in 1895 (1901). Note that Orkney and Shetland are combined. Otherwise, the model is as in Table 7. Two outliers (Banff and Clackmannan) are omitted from the graph. The line is fitted by locally estimated scatterplot smoothing. The labels in magenta are in the Highlands, Islands and North East; labels in orange are in the south.

The unidiff parameters were then graphed against the index of school‐attendance in Figure 4, omitting the two counties with values of the index greater than 100 (Banff and Clackmannan). Far from suggesting that relatively greater fluidity might be associated with relatively high levels of educational participation, the pattern is mostly the very opposite. The same conclusion was reached if, instead of the unidiff estimates, the vertical axis was any of the six topological parameters associated with mobility into the professional and managerial class from each of the other classes. For each of these, counties with higher levels of school attendance had lower rates of this upward mobility. There might then be a further possible explanation. Perhaps attending school was not only irrelevant: it was actually an impediment to fluidity if it took children away from early employment. Historical analysis has frequently noted that, when the state was trying to improve school attendance in the late‐nineteenth century, the main resistance from parents was to the loss of family income when older children could no longer enter the labour market (Anderson, 1995, pp. 229–236), especially because the schools did not themselves provide much vocational preparation. Vocational education in Britain at that time was weak compared to the rapidly industrialing new economies (notably Germany, the United States and Japan). Thus the route to advancement in employment (and thus to social mobility) depended on training in work, which in turn required not being in school.

## 6 CONCLUSIONS

Our paper has provided a comprehensive study of social mobility patterns in Scotland from the late 19^th^ century to the beginning of the 21^st^ century and linked these patterns to nation‐wide as well as local structural economic changes. We have addressed four questions. It appears that Scotland can be added to the accumulation of evidence that male social mobility in the late‐nineteenth century was extensive (question 2), and that it slowly grew even further in the following century (question 1). The rate of that growth was about 1.5 percentage points per decade (Table 1), though possibly slowing to 1 point per decade towards the end. These broad numbers are not dissimilar to those reported for England by Long (2013) and Lambert et al. (2007), as noted earlier. Thus patterns of social mobility in Scotland are not different from those found in England, despite the somewhat later industrialisation, the Scottish myth of opportunity, and the other differences noted at the beginning of the paper. The slow increase of mobility over the century contradicts the claims made in recent years that social mobility in Britain stagnated towards the end of the twentieth century (Blanden et al., 2004; Goldthorpe, 2013). Our evidence suggests that, at least in Scotland, mobility had never been greater since the high point of Victorian industrial society.

This mobility extended across the full range of social classes, as thoroughly in 1901 as later. Although stability in the same class as their father was very common for men aged 30–49 in 1901—and especially so for skilled manual workers—there was also much movement across classes that are widely separated in the hierarchy. Thus around one in five or six men whose fathers were in manual occupations in 1871 were themselves in the non‐manual or self‐employed classes in 1901. Moreover, because the highest‐status classes were relatively small, the origins of the people who occupied them were very diverse. Fully 57% of men in the salariat came from manual‐class origins. This was probably the basis of the persisting belief at the time that Scotland was a pioneering meritocracy.

Turning to our third question concerning the link between local occupational structures and mobility patterns we found that this relative openness in Scotland was associated with the decline of agriculture rather than with any more deliberate social intent. Most of the regionally distinctive patterns related to movement that involved agricultural employment (Figures 1, 2 and 3). Although, across Scotland as a whole, class inheritance—the tendency for sons to be in the same class as their father—was strongest in the agricultural classes, these classes in the predominantly agricultural areas were the source of more outward mobility than the other classes. In one sense, this variation by area was analogous to that found elsewhere, as noted above for the Netherlands and Hungary (Lippényi et al., 2015; Schulz et al., 2015), but, unlike there, social mobility was not greater in the more industrialised areas: it was associated in Scotland with areas that were still going through the transition from an agricultural economy, not with areas that had already reached a very high level of industrialisation. Lastly, despite the widely held Scottish belief that education was the key to social mobility, there was no evidence that higher rates of participation in the new national system of education after 1872 were associated with higher rates of mobility (question 4). Indeed, quite the opposite may have been the case (Figure 4), reminding us that the labour of adolescents remained essential to household finances. In particular, therefore, this tends to give support to our description of the analysis as being about period effects, not about cohorts, because cohort effects would be likely to be caused by, or associated with, changing patterns of educational participation. Furthermore, because we have standardised on the age range 30–49, our results are not influenced by changing patterns of opportunity experienced by different cohorts as they moved through their careers.

In short, the impression from the nineteenth‐century data and from the long‐term comparisons reinforces previous conclusions that social mobility was driven by broad economic change. The main factor was probably not educational expansion, although education became a mechanism by which the economy had its impact. Mobility was also probably not a consequence of the welfare state: social mobility was already a common experience of men long before the advent of state welfare in the twentieth century. These results do seem to be consistent not only with historians' more detailed analysis of local or individual experience, but also with contemporary commentary (as noted by Miles, 1993): mobility was simply a consequence of the massive social disruption that accompanied industrialism and modernity.

## Supporting information

Supporting Information S1

## Data Availability

The data that support the findings of this study are openly available in UK Data Archive at https://www.data‐archive.ac.uk/.

## ENDNOTES

^1^
The data (Schurer and Higgs, 2024) are available from the UK Data Service (study number 7856) under Special Licence due to commercial sensitivity (though there is no requirement to analyse them in a designated secure lab setting).

^2^
I‐CeM was a research programme funded by the Economic and Social Research Council (ESRC RES‐062‐23‐1629) between 2009 and 2013 which standardised the censuses of Great Britain for the period 1851–1911.

^3^
Wen et al. (2022) have also linked the 1861 and 1891 Scottish censuses and we have replicated the analyses concerning questions 1 and 2 using these data. The results are almost identical to those using the 1871–1901 linked data.

^4^
The index of dissimilarity is the proportion of men who would have to change their destination class to make the distributions of destinations and origins identical.

^5^
These results were robust to how many classes in the Goldthorpe scheme were used. Using three classes (non‐manual (I, II, III, IVa,b); manual (V, VI, VIIa); agricultural (IVc, VIIb)) gave unidiff estimates of −0.17 for 1974 and ‐0.35 for 2001. Omitting the two agricultural classes IVc and VIIb from the seven‐class scheme gave values of −0.17 and −0.38.
