# Supplementary material for: A long view of social mobility in Scotland and the role of economic changes
Source: Br J Sociol. 2024 Nov 17;76(2):241–59. doi: 10.1111/1468-4446.13162 (PMC11890437; doi:10.1111/1468-4446.13162)
Supplement: Supplementary file 1 — Supporting Information S1 [file BJOS-76-241-s001.docx]

**Supporting Information for**

**‘A long view of social mobility in Scotland and the role of economic changes’**

**Table S1: Loglinear models: analysis of deviance**

| *Terms in model* | | Df | Deviance | p |
| --- | --- | --- | --- | --- |
| 1 | All one-way | 10 | 255,906 | <0.001 |
| 2 | Origin by date | 8 | 2,157 | <0.001 |
| 3 | Destination by date | 8 | 2,548 | <0.001 |
| 4 | Origin by destination | 16 | 14,635 | <0.001 |
|  | Residual | 32 | 241 | <0.001 |
| 5 | Unidiff (date) | 2 | 58 | <0.001 |
|  | Residual | 30 | 183 | <0.001 |

Generalised linear model of table of origin-by-destination-by- date, with Poisson distribution and logarithmic link function.

The term shown for each of models 2, 3, 4 and 5 is added to the model immediately above it.

For all dates, in both origin and destination, ‘self-employed (agriculture)’ is grouped with ‘self-employed’, and ‘agricultural worker’ is grouped with ‘semi-skillled and unskilled’.

**Table S2: Standardised deviance residuals from the constant-association model in Table S1**

***1901***

|  | *Destination* | | | | |
| --- | --- | --- | --- | --- | --- |
| *Origin* | Professional and manager | Routine non-manual | Self-employed | Supervisor and skilled manual | Semi-skilled and unskilled manual |
| Professional and manager | -0.2 | 0.4 | -0.3 | 0.3 | -0.1 |
| Routine non-manual | 0.9 | 1.9 | 0.4 | -1.4 | -1.0 |
| Self-employed | -0.1 | 0.1 | 0.0 | -0.3 | 0.3 |
| Supervisor and skilled manual | -0.9 | -0.2 | -0.1 | 0.9 | -0.8 |
| Semi-skilled and unskilled manual | 0.9 | -0.8 | 0.2 | -0.9 | 0.6 |

***1974***

|  | *Destination* | | | | |
| --- | --- | --- | --- | --- | --- |
| *Origin* | Professional and manager | Routine non-manual | Self-employed | Supervisor and skilled manual | Semi-skilled and unskilled manual |
| Professional and manager | 1.3 | -1.3 | -0.6 | 0.8 | -2.3 |
| Routine non-manual | -0.5 | -2.3 | -1.4 | 2.0 | 1.4 |
| Self-employed | 0.5 | -2.0 | 2.1 | 0.2 | -1.9 |
| Supervisor and skilled manual | 0.8 | -0.3 | 0.1 | -3.7 | 4.5 |
| Semi-skilled and unskilled manual | -2.2 | 3.5 | -1.4 | 3.6 | -2.5 |

***2001***

|  | *Destination* | | | | |
| --- | --- | --- | --- | --- | --- |
| *Origin* | Professional and manager | Routine non-manual | Self-employed | Supervisor and skilled manual | Semi-skilled and unskilled manual |
| Professional and manager | -0.3 | -0.5 | 2.0 | -1.7 | 2.3 |
| Routine non-manual | -0.8 | -3.0 | -0.4 | 2.5 | 2.1 |
| Self-employed | -0.1 | 1.4 | -2.5 | 2.9 | -2.4 |
| Supervisor and skilled manual | 2.7 | 2.1 | 1.6 | -4.9 | 2.1 |
| Semi-skilled and unskilled manual | -1.3 | 0.9 | -0.6 | 3.4 | -2.9 |

These are the standardised deviance residuals from Model 4 in Table S1.

**Table S3: Parameter estimates from model of class inheritance**

|  | Parameter estimates (differences from 1901 values) | | | |
| --- | --- | --- | --- | --- |
| Class | 1974 | s.e. | 2001 | s.e. |
| Professional and manager | 0.57 | 0.18 | 0.16 | 0.13 |
| Routine non-manual | -0.95 | 0.37 | -0.73 | 0.25 |
| Self-employed | 0.69 | 0.20 | -0.39 | 0.27 |
| Supervisor and skilled manual | -0.71 | 0.12 | -0.95 | 0.14 |
| Semi-skilled and unskilled manual | -0.30 | 0.12 | -0.38 | 0.16 |

**Table S4: Employment (1901) and educational (1895) characteristics of counties**

| County of residence | Size in data set | Region | Percentage male employment in ‘agriculture and fisheries’ (1901) | Percentage male employment in ‘industry’ (1901) | Index of school attendance (1895) |
| --- | --- | --- | --- | --- | --- |
| All Scotland |  |  | 12 | 53 | 86 |
| Aberdeen | 6,388 | North East | 24 | 40 | 95 |
| Angus | 5,857 | North East | 10 | 55 | 87 |
| Argyll | 1,109 | Highlands & Islands | 30 | 32 | 80 |
| Ayr | 5,815 | West Central | 9 | 60 | 89 |
| Banff | 1,165 | North East | 42 | 27 | 102 |
| Berwick | 910 | South | 41 | 27 | 79 |
| Bute | 326 | Highlands & Islands | 22 | 34 | 74 |
| Caithness | 734 | Highlands & Islands | 41 | 28 | 76 |
| Clackmannan | 602 | North Central | 5 | 64 | 113 |
| Dumfries | 1,691 | South | 25 | 38 | 91 |
| Dunbarton | 1,825 | West Central | 5 | 63 | 94 |
| East Lothian | 955 | East Central | 30 | 38 | 86 |
| Fife | 5,530 | North Central | 10 | 58 | 97 |
| Inverness | 968 | Highlands & Islands | 34 | 26 | 77 |
| Kincardine | 1,056 | North East | 40 | 28 | 91 |
| Kinross | 187 | North Central | 28 | 40 | 76 |
| Kirkcudbright | 772 | South | 29 | 35 | 92 |
| Lanark | 22,345 | West Central | 2 | 65 | 86 |
| Midlothian | 7,794 | East Central | 3 | 52 | 78 |
| Moray | 619 | North East | 32 | 33 | 98 |
| Nairn | 140 | North East | 39 | 30 | 70 |
| Orkney | 825 | Highlands & Islands | 56 | 18 | - |
| Peebles | 364 | South | 22 | 45 | 81 |
| Perth | 2,637 | North Central | 23 | 38 | 78 |
| Renfrew | 4,921 | West Central | 3 | 62 | 82 |
| Ross & Cromarty | 941 | Highlands & Islands | 49 | 19 | 73 |
| Roxburgh | 1,326 | South | 24 | 41 | 82 |
| Selkirk | 597 | South | 11 | 59 | 91 |
| Shetland | 599 | Highlands & Islands | 50 | 19 |  |
| Stirling | 2,869 | North Central | 6 | 60 | 88 |
| Sutherland | 309 | Highlands & Islands | 49 | 22 | 73 |
| West Lothian | 1,334 | East Central | 6 | 66 | 94 |
| Wigtown | 547 | South | 37 | 46 | 84 |
| Orkney and Shetland | 1,424 | Highlands and Islands | - | - | 70 |

Source: The sample sizes are in the analysed data set. In these data, the cities were included with the counties: Aberdeen is in Aberdeen county, Dundee in Angus, Edinburgh in Midlothian, and Glasgow in Lanark. Some other counties have been given post-1920s names here, for ease of recognition (for example East Lothian for what was officially the county of Haddington). Thus this list covers the whole of Scotland, despite the implications of some of the names.

The employment percentages come from Table VI in Eleventh Decennial Census of the Population of Scotland Taken 31st March 1901, with Report. Vol. III. The educational percentages come from the annual report of the Scotch Education Department (1896). In that report, Orkney and Shetland are grouped together and so are also shown grouped here.

**Figure S1: Distribution of standardised deviance residuals from the Common Social Fluidity model in Table 6**


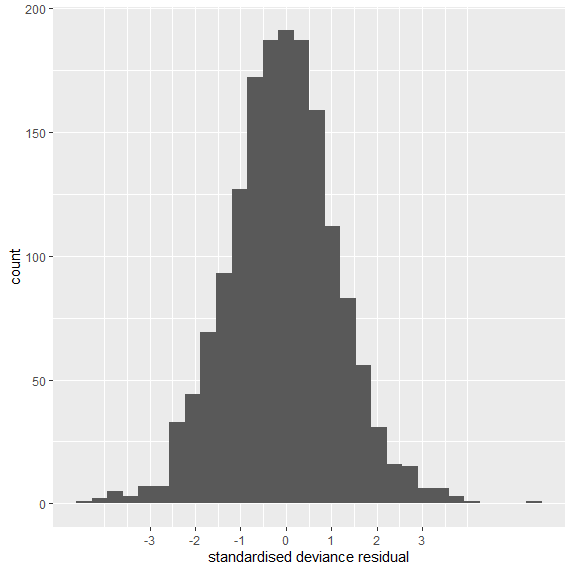


The residuals from the Common Social Fluidity model in Table 6 correspond to 7x7 tables of origin-by-destination in each of 33 counties.

**Table S5: Standardised deviance residuals greater than 1.96 from the Common Social Fluidity model in Table 6**

**Out of agriculture**

| *Origin class* | *Destination class* | *County* | *Residual* |
| --- | --- | --- | --- |
| Self-employed (agriculture) | Professional or manager | Fife | 2.1 |
| Self-employed (agriculture) | Professional or manager | Midlothian | 2.5 |
| Self-employed (agriculture) | Routine non-manual | East Lothian | 2.1 |
| Self-employed (agriculture) | Supervisor and skilled manual | Aberdeen | 2.3 |
| Self-employed (agriculture) | Supervisor and skilled manual | Argyll | 2.9 |
| Self-employed (agriculture) | Supervisor and skilled manual | Banff | 3.0 |
| Self-employed (agriculture) | Supervisor and skilled manual | Caithness | 4.0 |
| Self-employed (agriculture) | Supervisor and skilled manual | Kincardine | 3.0 |
| Self-employed (agriculture) | Supervisor and skilled manual | Orkney | 3.0 |
| Self-employed (agriculture) | Supervisor and skilled manual | Ross and Cromarty | 2.7 |
| Self-employed (agriculture) | Supervisor and skilled manual | Sutherland | 2.7 |
| Self-employed (agriculture) | Semi-skilled and unskilled manual | Bute | 2.1 |
| Self-employed (agriculture) | Semi-skilled and unskilled manual | Kinross | 2.2 |
| Self-employed (agriculture) | Semi-skilled and unskilled manual | Shetland | 2.8 |
| Agricultural worker | Professional or manager | Lanark | 3.5 |
| Agricultural worker | Routine non-manual | Lanark | 3.7 |
| Agricultural worker | Self-employed (not agriculture) | Lanark | 2.0 |
| Agricultural worker | Supervisor and skilled manual | Shetland | 2.1 |
| Agricultural worker | Semi-skilled and unskilled manual | Kirkcudbright | 2.0 |
| Agricultural worker | Semi-skilled and unskilled manual | Stirling | 2.2 |

**Into agriculture**

| Professional or manager | Agricultural worker | Kinross | 3.0 |
| --- | --- | --- | --- |
| Routine non-manual | Agricultural worker | Banff | 2.1 |
| Routine non-manual | Agricultural worker | Roxburgh | 2.1 |
| Supervisor and skilled manual | Self-employed (agriculture) | Aberdeen | 2.7 |
| Supervisor and skilled manual | Self-employed (agriculture) | Caithness | 2.3 |
| Supervisor and skilled manual | Self-employed (agriculture) | Inverness | 3.9 |
| Supervisor and skilled manual | Self-employed (agriculture) | Kincardine | 2.4 |
| Supervisor and skilled manual | Self-employed (agriculture) | Midlothian | 2.5 |
| Supervisor and skilled manual | Self-employed (agriculture) | Orkney | 2.4 |
| Supervisor and skilled manual | Self-employed (agriculture) | Ross and Cromarty | 2.9 |
| Supervisor and skilled manual | Self-employed (agriculture) | Shetland | 3.8 |
| Supervisor and skilled manual | Self-employed (agriculture) | Sutherland | 2.6 |
| Supervisor and skilled manual | Agricultural worker | Inverness | 2.4 |
| Supervisor and skilled manual | Agricultural worker | Perth | 2.4 |
| Semi-skilled and unskilled manual | Self-employed (agriculture) | Banff | 2.4 |
| Semi-skilled and unskilled manual | Self-employed (agriculture) | Inverness | 2.8 |
| Semi-skilled and unskilled manual | Self-employed (agriculture) | Ross And Cromarty | 3.4 |
| Semi-skilled and unskilled manual | Agricultural worker | Argyll | 2.7 |
| Semi-skilled and unskilled manual | Agricultural worker | Kirkcudbright | 2.0 |
| Semi-skilled and unskilled manual | Agricultural worker | Midlothian | 2.1 |
| Semi-skilled and unskilled manual | Agricultural worker | Orkney | 2.2 |
| Semi-skilled and unskilled manual | Agricultural worker | Ross And Cromarty | 2.1 |
| Semi-skilled and unskilled manual | Agricultural worker | Stirling | 2.2 |

**Between and within agricultural classes**

| Self-employed (agriculture) | Self-employed (agriculture) | Ayr | 3.5 |
| --- | --- | --- | --- |
| Self-employed (agriculture) | Self-employed (agriculture) | Renfrew | 3.3 |
| Self-employed (agriculture) | Self-employed (agriculture) | Stirling | 2.1 |
| Self-employed (agriculture) | Agricultural worker | Ayr | 2.6 |
| Self-employed (agriculture) | Agricultural worker | Clackmannan | 2.3 |
| Self-employed (agriculture) | Agricultural worker | Dunbarton | 3.6 |
| Self-employed (agriculture) | Agricultural worker | Lanark | 3.0 |
| Self-employed (agriculture) | Agricultural worker | Shetland | 2.7 |
| Agricultural worker | Self-employed (agriculture) | Angus | 2.4 |
| Agricultural worker | Self-employed (agriculture) | Midlothian | 2.3 |
| Agricultural worker | Self-employed (agriculture) | Orkney | 2.7 |
| Agricultural worker | Self-employed (agriculture) | Shetland | 5.6 |
| Agricultural worker | Agricultural worker | Angus | 2.0 |
| Agricultural worker | Agricultural worker | Ayr | 2.2 |
| Agricultural worker | Agricultural worker | Fife | 3.4 |

**Not agriculture: same class as father**

| Self-employed (not agriculture) | Self-employed (not agriculture) | Argyll | 3.0 |
| --- | --- | --- | --- |
| Self-employed (not agriculture) | Self-employed (not agriculture) | Ayr | 2.2 |
| Self-employed (not agriculture) | Self-employed (not agriculture) | Kirkcudbright | 2.7 |
| Supervisor and skilled manual | Supervisor and skilled manual | East Lothian | 2.0 |
| Supervisor and skilled manual | Supervisor and skilled manual | Fife | 2.1 |

**Not agriculture: different class from father**

| Professional or manager | Routine non-manual | Dunbarton | 2.3 |
| --- | --- | --- | --- |
| Professional or manager | Supervisor and skilled manual | Angus | 2.0 |
| Professional or manager | Supervisor and skilled manual | Lanark | 2.6 |
| Professional or manager | Semi-skilled and unskilled manual | Fife | 2.5 |
| Self-employed (not agriculture) | Routine non-manual | Shetland | 2.1 |
| Self-employed (not agriculture) | Semi-skilled and unskilled manual | Lanark | 2.2 |
| Supervisor and skilled manual | Professional or manager | Orkney | 2.2 |
| Supervisor and skilled manual | Routine non-manual | Angus | 2.7 |
| Semi-skilled and unskilled manual | Self-employed (not agriculture) | Fife | 2.6 |
| Semi-skilled and unskilled manual | Supervisor and skilled manual | Renfrew | 2.0 |

These are all the positive residuals greater than 1.96 from the Common Social Fluidity model in Table 6.
